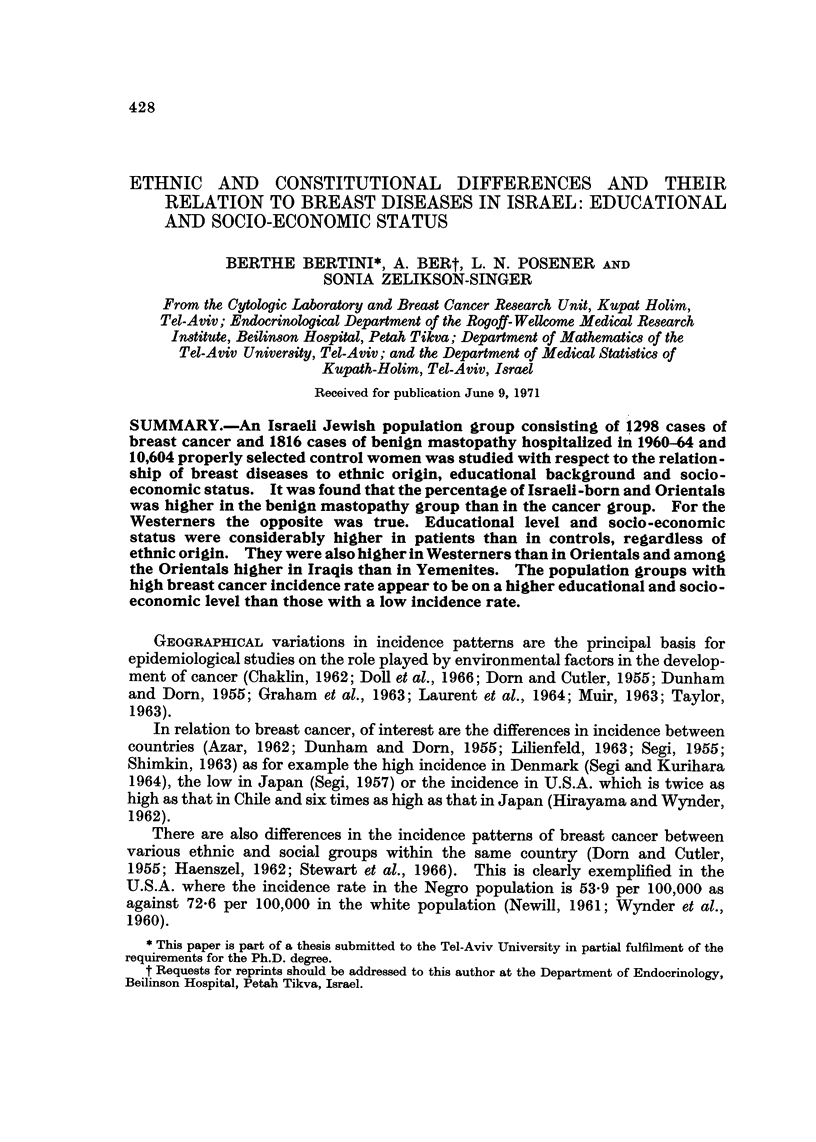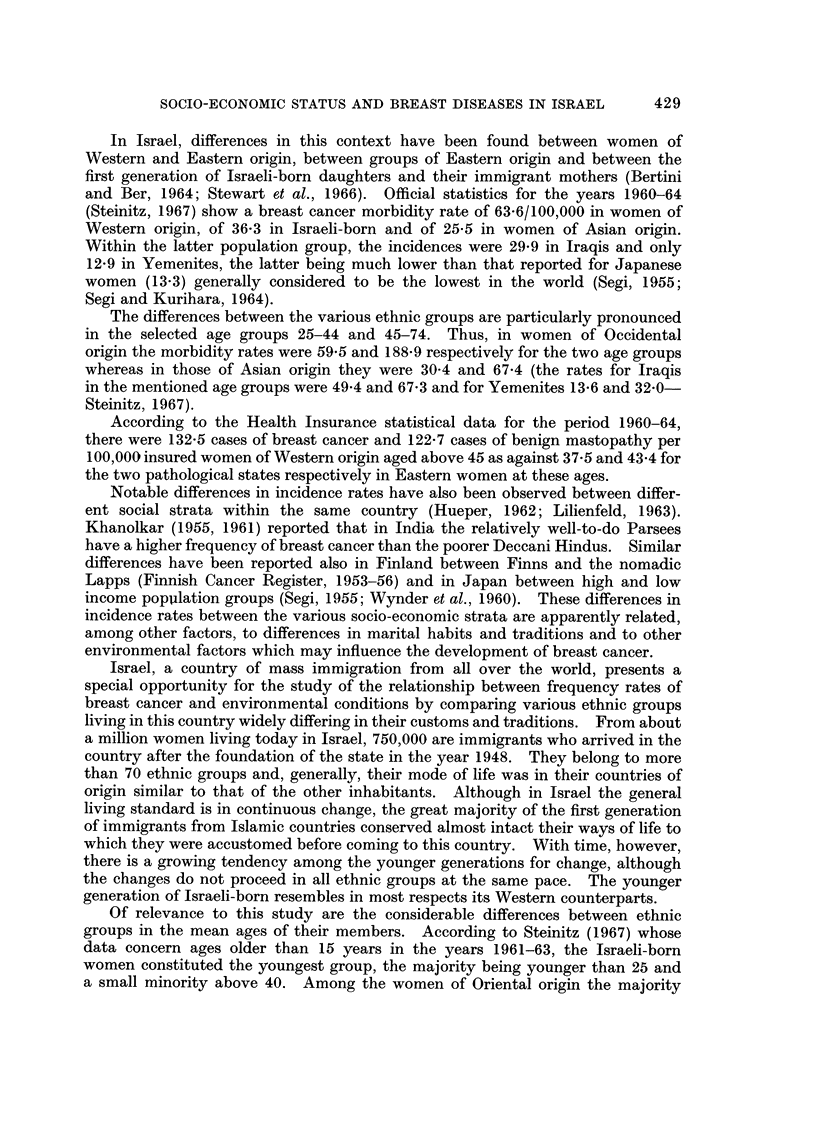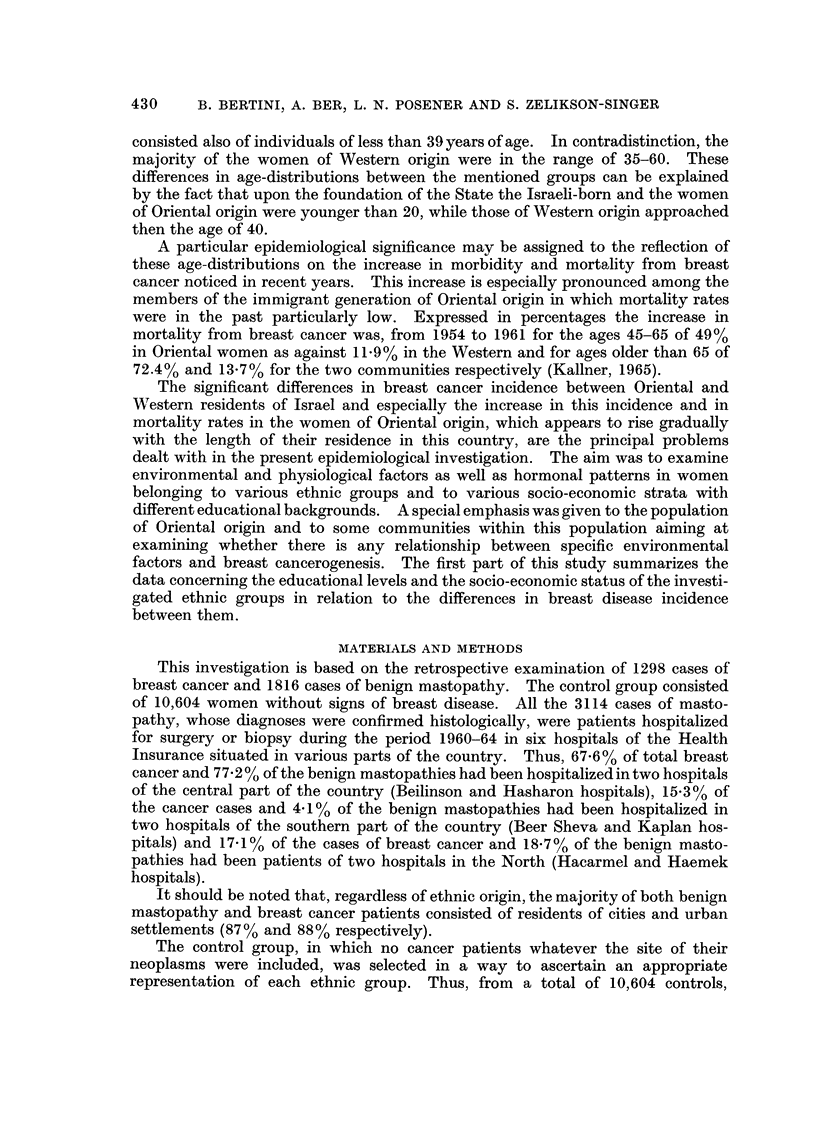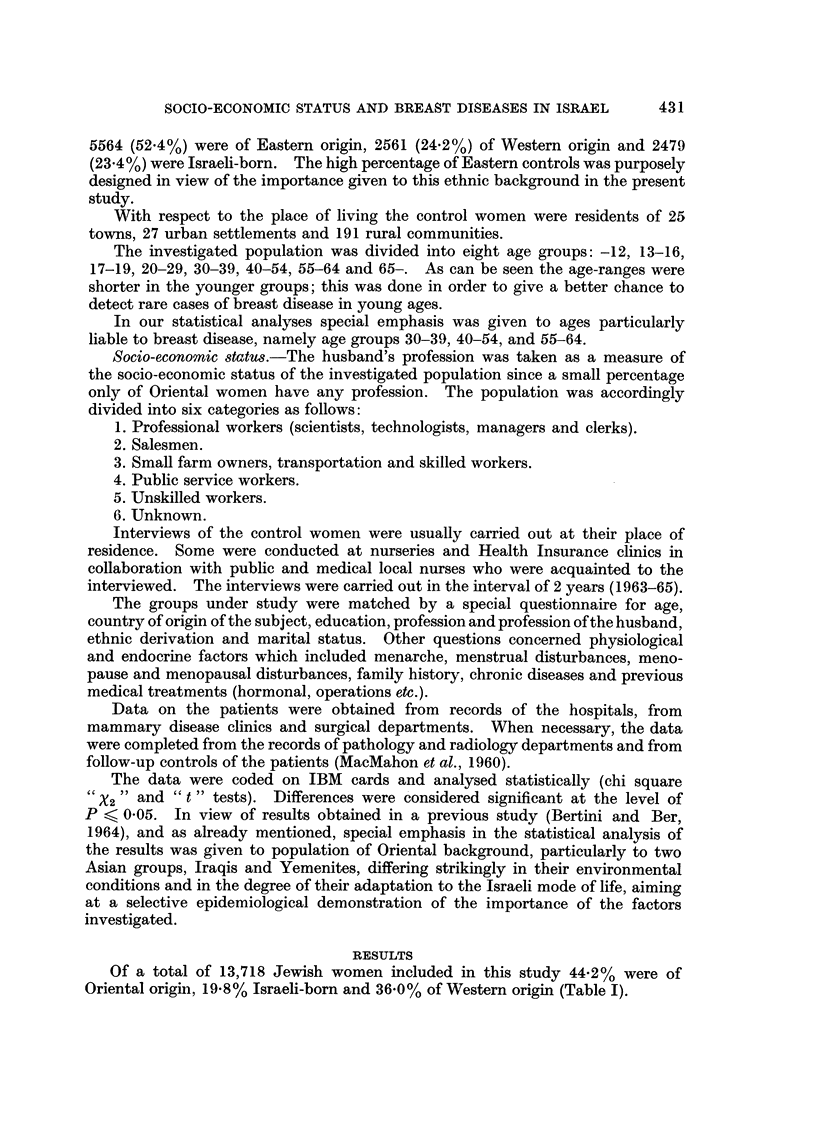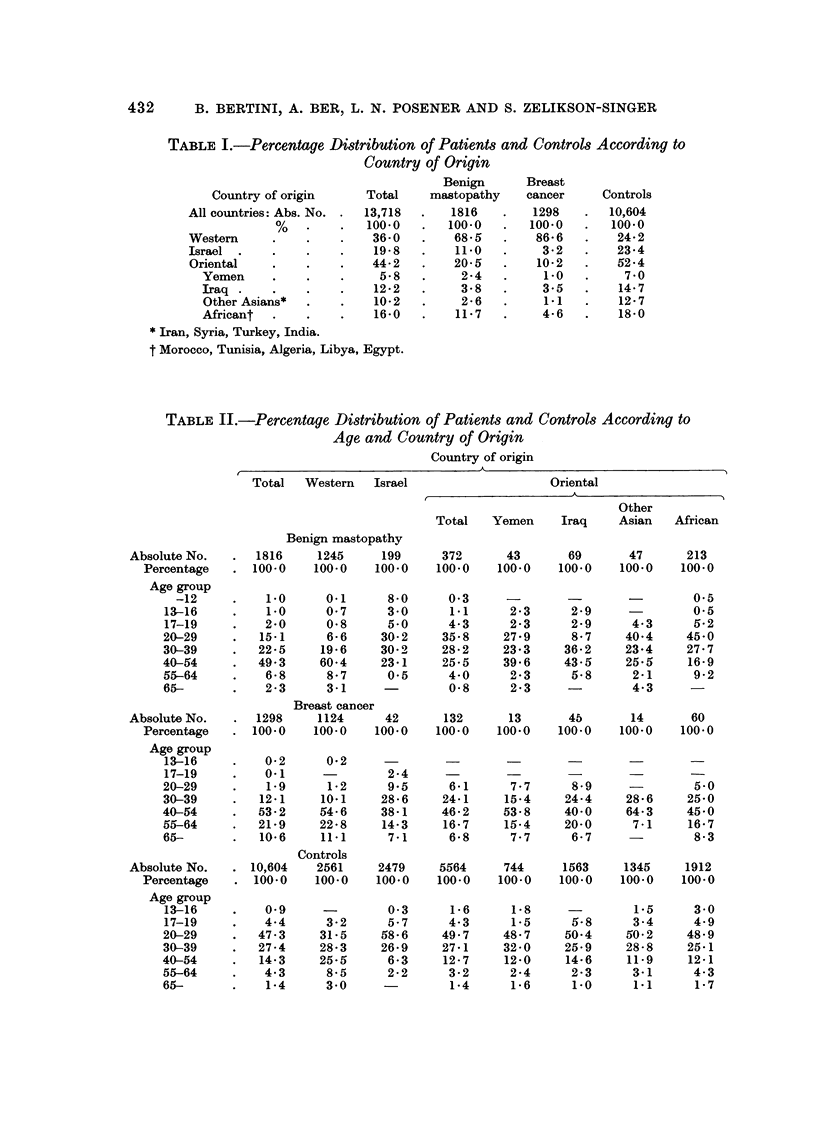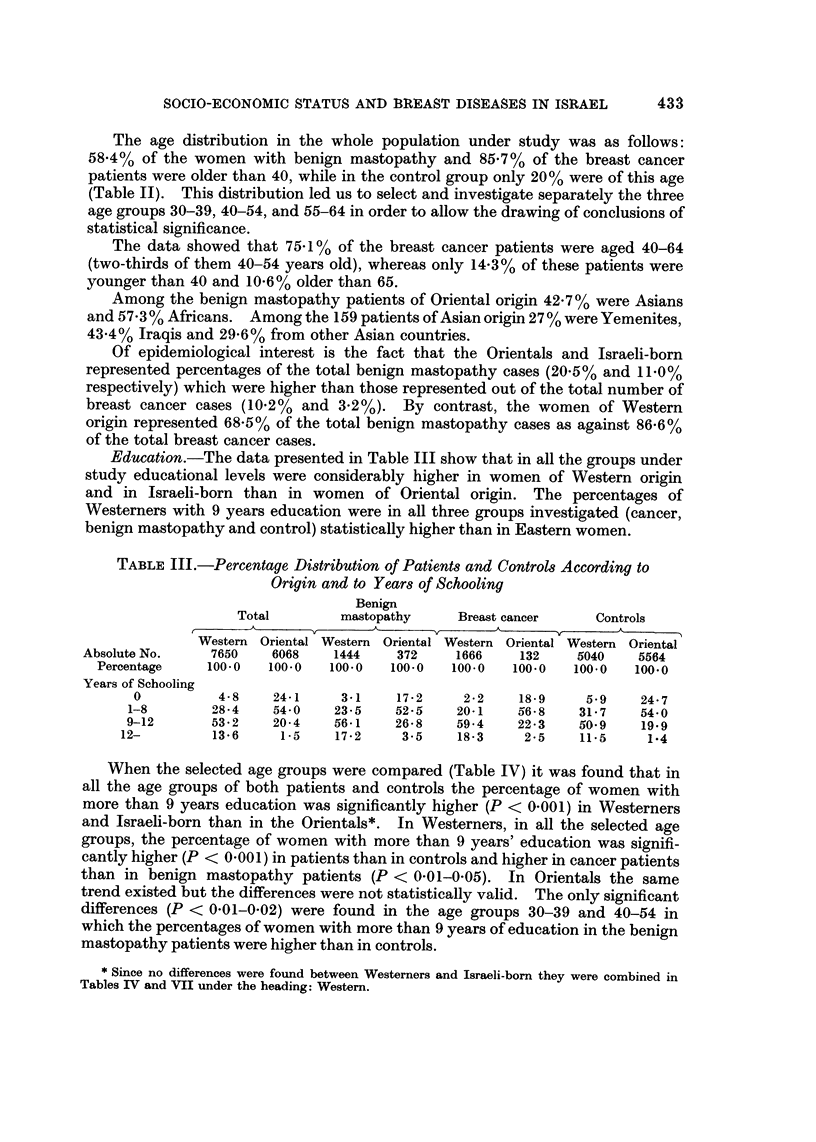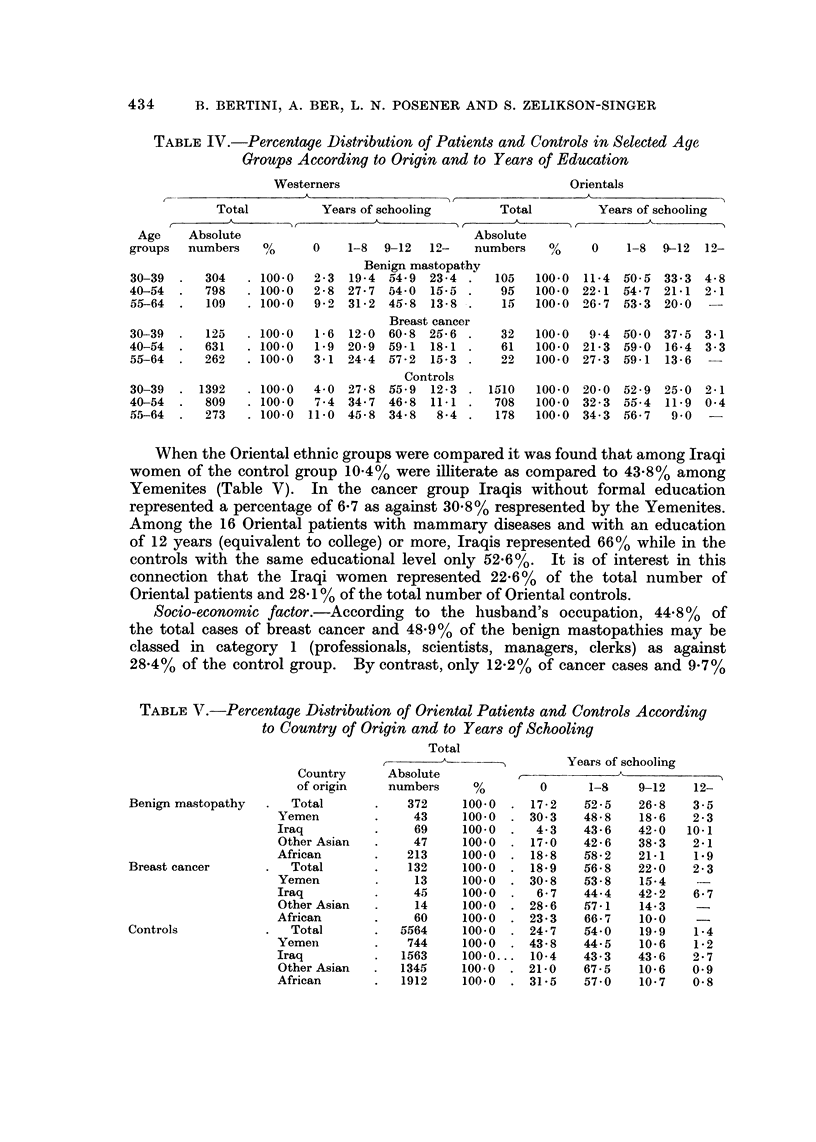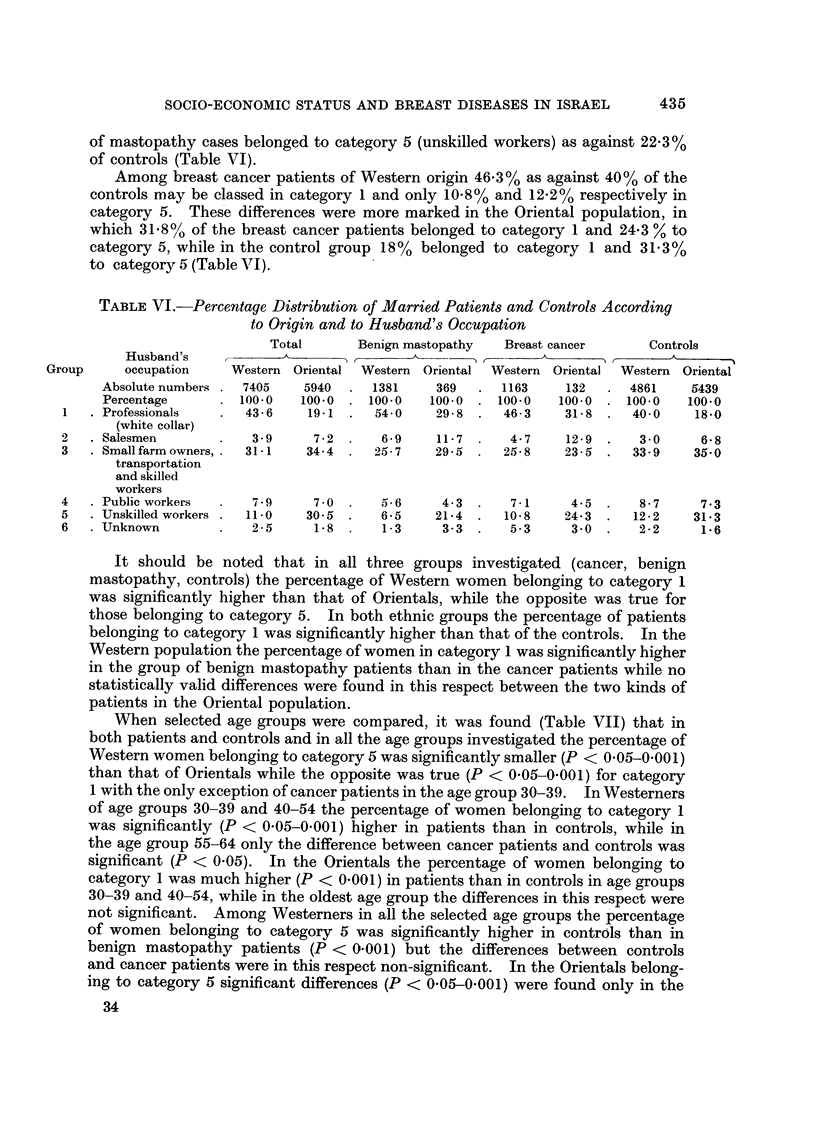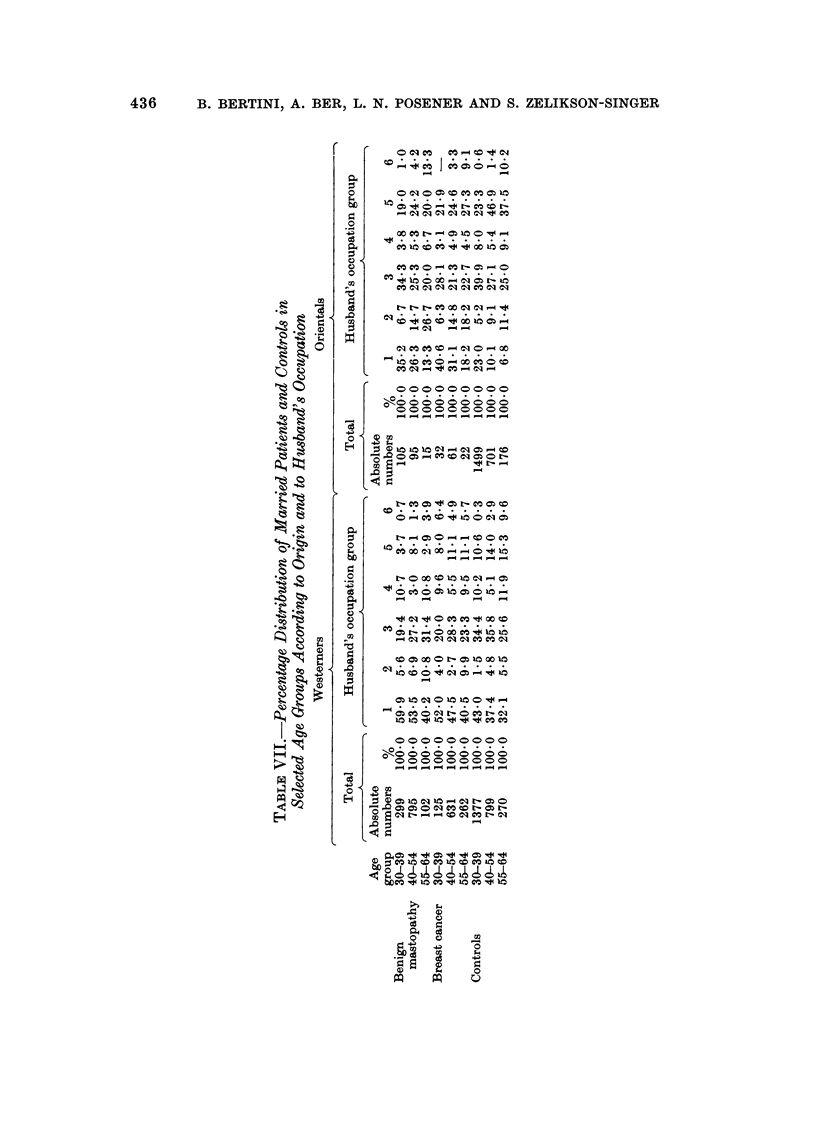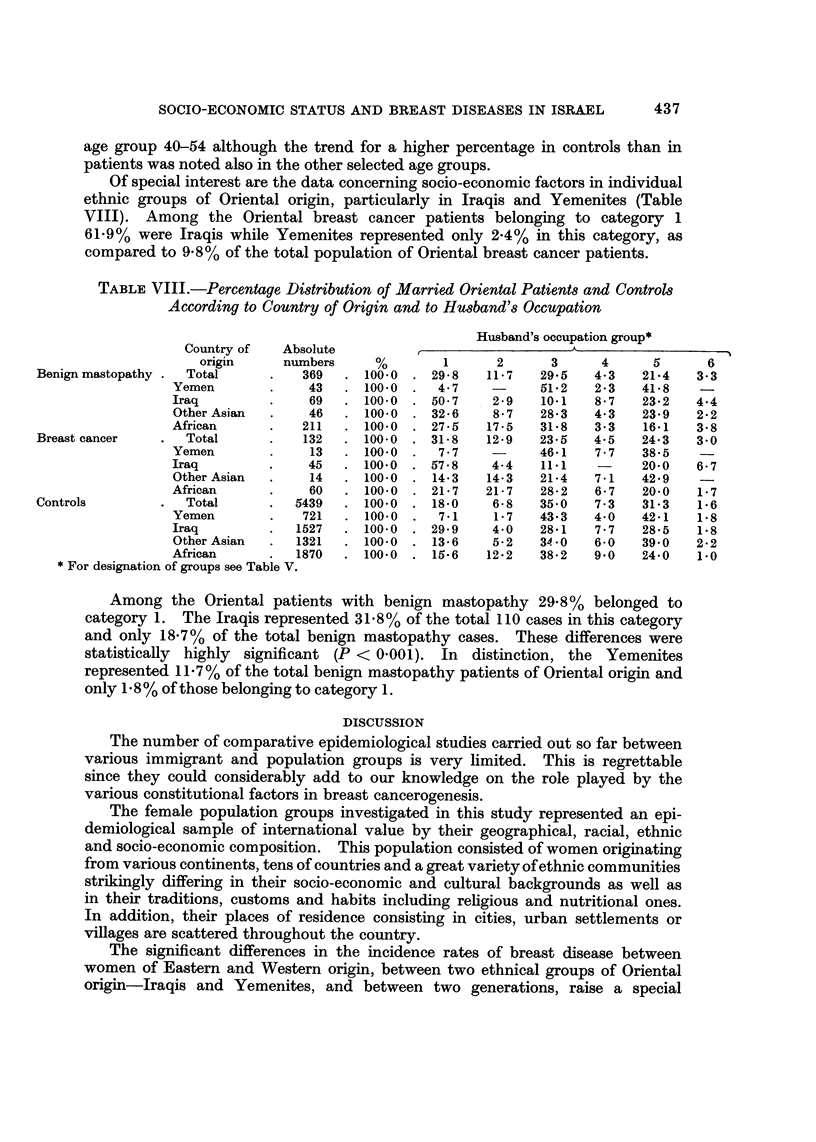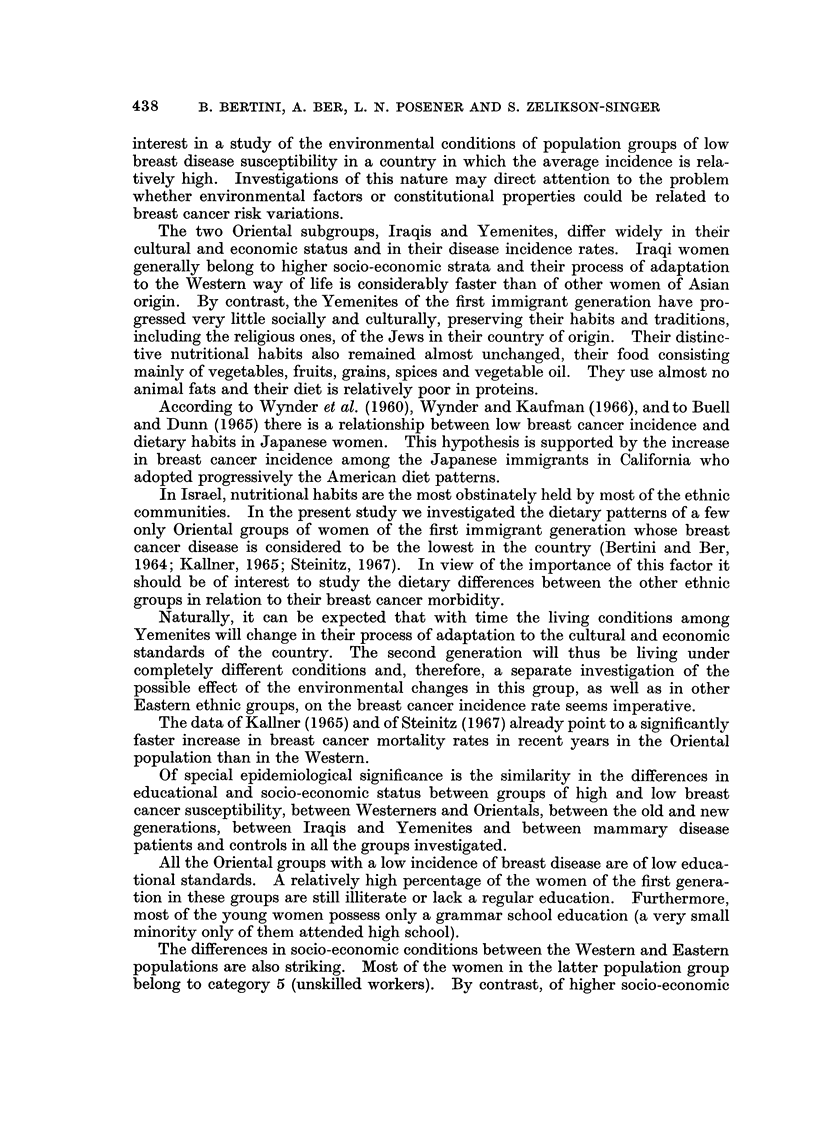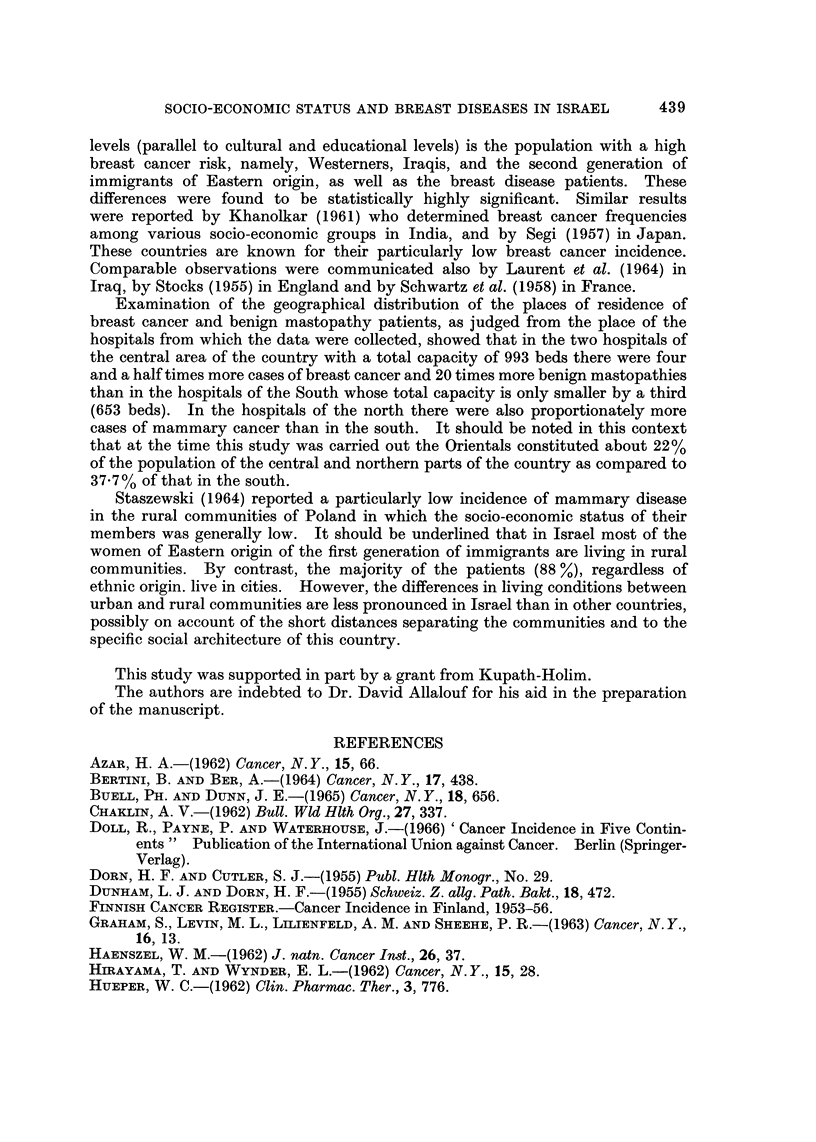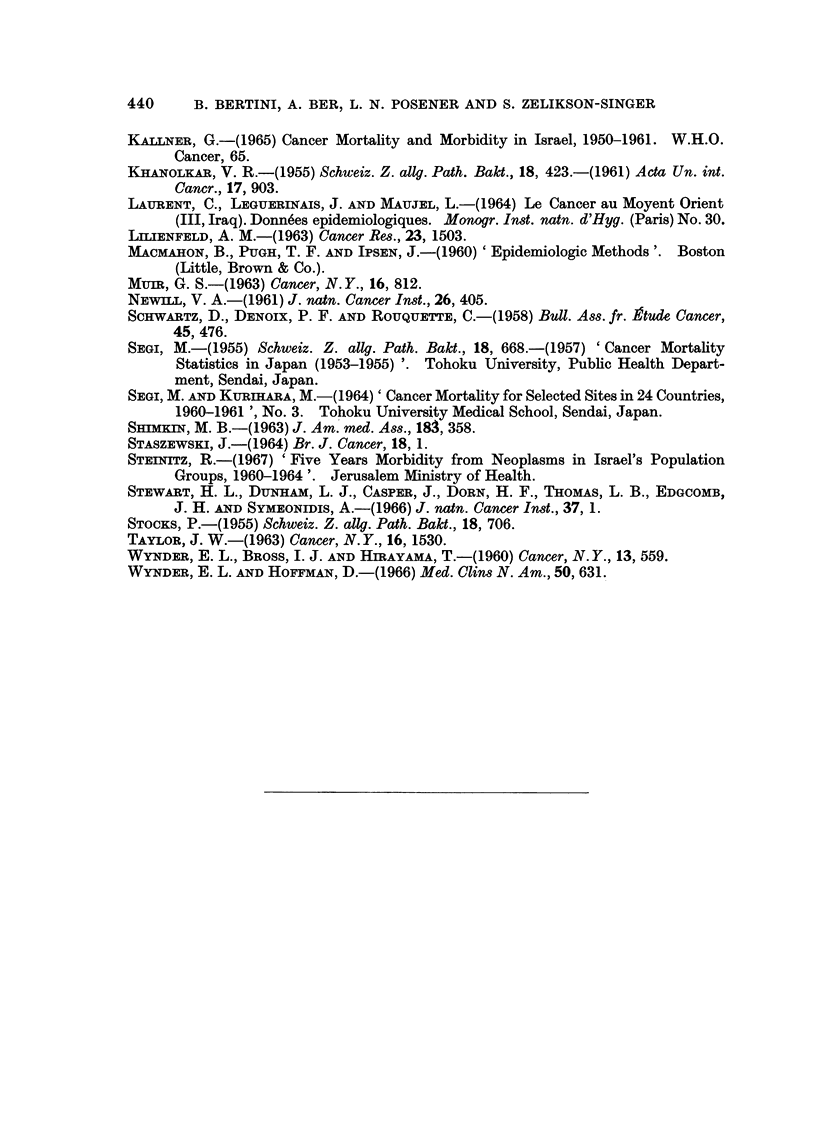# Ethnic and Constitutional Differences and their Relation to Breast Diseases in Israel: Educational and Socio-Economic Status

**DOI:** 10.1038/bjc.1971.55

**Published:** 1971-09

**Authors:** Berthe Bertini, A. Ber, L. N. Posener, Sonia Zelikson-Singer

## Abstract

An Israeli Jewish population group consisting of 1298 cases of breast cancer and 1816 cases of benign mastopathy hospitalized in 1960-64 and 10,604 properly selected control women was studied with respect to the relationship of breast diseases to ethnic origin, educational background and socio-economic status. It was found that the percentage of Israeli-born and Orientals was higher in the benign mastopathy group than in the cancer group. For the Westerners the opposite was true. Educational level and socio-economic status were considerably higher in patients than in controls, regardless of ethnic origin. They were also higher in Westerners than in Orientals and among the Orientals higher in Iraqis than in Yemenites. The population groups with high breast cancer incidence rate appear to be on a higher educational and socio-economic level than those with a low incidence rate.


### ETHNIC AND CONSTITUTIONAL DIFFERENCES AND THEIR RELATION TO BREAST DISEASES IN ISRAEL: EDUCATIONAL AND SOCIO-ECONOMIC STATUS

BERTHE BERTINI*, A. BER†, L. N. POSENER AND SONIA ZELIKSON-SINGER

*From the Cytologic Laboratory and Breast Cancer Research Unit, Kupat Holim, Tel-Aviv; Endocrinological Department of the Rogoff-Wellcome Medical Research Institute, Beilinson Hospital, Petah Tikva; Department of Mathematics of the Tel-Aviv University, Tel-Aviv; and the Department of Medical Statistics of Kupath-Holim, Tel-Aviv, Israel*

Received for publication June 9, 1971

**SUMMARY.—An Israeli Jewish population group consisting of 1298 cases of breast cancer and 1816 cases of benign mastopathy hospitalized in 1960–64 and 10,604 properly selected control women was studied with respect to the relationship of breast diseases to ethnic origin, educational background and socio-economic status. It was found that the percentage of Israeli-born and Orientals was higher in the benign mastopathy group than in the cancer group. For the Westerners the opposite was true. Educational level and socio-economic status were considerably higher in patients than in controls, regardless of ethnic origin. They were also higher in Westerners than in Orientals and among the Orientals higher in Iraqis than in Yemenites. The population groups with high breast cancer incidence rate appear to be on a higher educational and socio-economic level than those with a low incidence rate.**

GEOGRAPHICAL variations in incidence patterns are the principal basis for epidemiological studies on the role played by environmental factors in the development of cancer (Chaklin, 1962; Doll *et al.*, 1966; Dorn and Cutler, 1955; Dunham and Dorn, 1955; Graham *et al.*, 1963; Laurent *et al.*, 1964; Muir, 1963; Taylor, 1963).

In relation to breast cancer, of interest are the differences in incidence between countries (Azar, 1962; Dunham and Dorn, 1955; Lilienfeld, 1963; Segi, 1955; Shimkin, 1963) as for example the high incidence in Denmark (Segi and Kurihara 1964), the low in Japan (Segi, 1957) or the incidence in U.S.A. which is twice as high as that in Chile and six times as high as that in Japan (Hirayama and Wynder, 1962).

There are also differences in the incidence patterns of breast cancer between various ethnic and social groups within the same country (Dorn and Cutler, 1955; Haenszel, 1962; Stewart *et al.*, 1966). This is clearly exemplified in the U.S.A. where the incidence rate in the Negro population is 53·9 per 100,000 as against 72·6 per 100,000 in the white population (Newill, 1961; Wynder *et al.*, 1960).

* This paper is part of a thesis submitted to the Tel-Aviv University in partial fulfilment of the requirements for the Ph.D. degree.

† Requests for reprints should be addressed to this author at the Department of Endocrinology, Beilinson Hospital, Petah Tikva, Israel.

In Israel, differences in this context have been found between women of Western and Eastern origin, between groups of Eastern origin and between the first generation of Israeli-born daughters and their immigrant mothers (Bertini and Ber, 1964; Stewart *et al.*, 1966). Official statistics for the years 1960–64 (Steinitz, 1967) show a breast cancer morbidity rate of 63·6/100,000 in women of Western origin, of 36·3 in Israeli-born and of 25·5 in women of Asian origin. Within the latter population group, the incidences were 29·9 in Iraqis and only 12·9 in Yemenites, the latter being much lower than that reported for Japanese women (13·3) generally considered to be the lowest in the world (Segi, 1955; Segi and Kurihara, 1964).

The differences between the various ethnic groups are particularly pronounced in the selected age groups 25–44 and 45–74. Thus, in women of Occidental origin the morbidity rates were 59·5 and 188·9 respectively for the two age groups whereas in those of Asian origin they were 30·4 and 67·4 (the rates for Iraqis in the mentioned age groups were 49·4 and 67·3 and for Yemenites 13·6 and 32·0—Steinitz, 1967).

According to the Health Insurance statistical data for the period 1960–64, there were 132·5 cases of breast cancer and 122·7 cases of benign mastopathy per 100,000 insured women of Western origin aged above 45 as against 37·5 and 43·4 for the two pathological states respectively in Eastern women at these ages.

Notable differences in incidence rates have also been observed between different social strata within the same country (Hueper, 1962; Lilienfeld, 1963). Khanolkar (1955, 1961) reported that in India the relatively well-to-do Parsees have a higher frequency of breast cancer than the poorer Deccani Hindus. Similar differences have been reported also in Finland between Finns and the nomadic Lapps (Finnish Cancer Register, 1953–56) and in Japan between high and low income population groups (Segi, 1955; Wynder *et al.*, 1960). These differences in incidence rates between the various socio-economic strata are apparently related, among other factors, to differences in marital habits and traditions and to other environmental factors which may influence the development of breast cancer.

Israel, a country of mass immigration from all over the world, presents a special opportunity for the study of the relationship between frequency rates of breast cancer and environmental conditions by comparing various ethnic groups living in this country widely differing in their customs and traditions. From about a million women living today in Israel, 750,000 are immigrants who arrived in the country after the foundation of the state in the year 1948. They belong to more than 70 ethnic groups and, generally, their mode of life was in their countries of origin similar to that of the other inhabitants. Although in Israel the general living standard is in continuous change, the great majority of the first generation of immigrants from Islamic countries conserved almost intact their ways of life to which they were accustomed before coming to this country. With time, however, there is a growing tendency among the younger generations for change, although the changes do not proceed in all ethnic groups at the same pace. The younger generation of Israeli-born resembles in most respects its Western counterparts.

Of relevance to this study are the considerable differences between ethnic groups in the mean ages of their members. According to Steinitz (1967) whose data concern ages older than 15 years in the years 1961–63, the Israeli-born women constituted the youngest group, the majority being younger than 25 and a small minority above 40. Among the women of Oriental origin the majority consisted also of individuals of less than 39 years of age. In contradistinction, the majority of the women of Western origin were in the range of 35–60. These differences in age-distributions between the mentioned groups can be explained by the fact that upon the foundation of the State the Israeli-born and the women of Oriental origin were younger than 20, while those of Western origin approached then the age of 40.

A particular epidemiological significance may be assigned to the reflection of these age-distributions on the increase in morbidity and mortality from breast cancer noticed in recent years. This increase is especially pronounced among the members of the immigrant generation of Oriental origin in which mortality rates were in the past particularly low. Expressed in percentages the increase in mortality from breast cancer was, from 1954 to 1961 for the ages 45–65 of 49% in Oriental women as against 11·9% in the Western and for ages older than 65 of 72.4% and 13·7% for the two communities respectively (Kallner, 1965).

The significant differences in breast cancer incidence between Oriental and Western residents of Israel and especially the increase in this incidence and in mortality rates in the women of Oriental origin, which appears to rise gradually with the length of their residence in this country, are the principal problems dealt with in the present epidemiological investigation. The aim was to examine environmental and physiological factors as well as hormonal patterns in women belonging to various ethnic groups and to various socio-economic strata with different educational backgrounds. A special emphasis was given to the population of Oriental origin and to some communities within this population aiming at examining whether there is any relationship between specific environmental factors and breast cancerogenesis. The first part of this study summarizes the data concerning the educational levels and the socio-economic status of the investigated ethnic groups in relation to the differences in breast disease incidence between them.

### MATERIALS AND METHODS

This investigation is based on the retrospective examination of 1298 cases of breast cancer and 1816 cases of benign mastopathy. The control group consisted of 10,604 women without signs of breast disease. All the 3114 cases of mastopathy, whose diagnoses were confirmed histologically, were patients hospitalized for surgery or biopsy during the period 1960–64 in six hospitals of the Health Insurance situated in various parts of the country. Thus, 67·6% of total breast cancer and 77·2% of the benign mastopathies had been hospitalized in two hospitals of the central part of the country (Beilinson and Hasharon hospitals), 15·3% of the cancer cases and 4·1% of the benign mastopathies had been hospitalized in two hospitals of the southern part of the country (Beer Sheva and Kaplan hospitals) and 17·1% of the cases of breast cancer and 18·7% of the benign mastopathies had been patients of two hospitals in the North (Hacarmel and Haemek hospitals).

It should be noted that, regardless of ethnic origin, the majority of both benign mastopathy and breast cancer patients consisted of residents of cities and urban settlements (87% and 88% respectively).

The control group, in which no cancer patients whatever the site of their neoplasms were included, was selected in a way to ascertain an appropriate representation of each ethnic group. Thus, from a total of 10,604 controls,

5564 (52·4%) were of Eastern origin, 2561 (24·2%) of Western origin and 2479 (23·4%) were Israeli-born. The high percentage of Eastern controls was purposely designed in view of the importance given to this ethnic background in the present study.

With respect to the place of living the control women were residents of 25 towns, 27 urban settlements and 191 rural communities.

The investigated population was divided into eight age groups: –12, 13–16, 17–19, 20–29, 30–39, 40–54, 55–64 and 65–. As can be seen the age-ranges were shorter in the younger groups; this was done in order to give a better chance to detect rare cases of breast disease in young ages.

In our statistical analyses special emphasis was given to ages particularly liable to breast disease, namely age groups 30–39, 40–54, and 55–64.

*Socio-economic status.*—The husband's profession was taken as a measure of the socio-economic status of the investigated population since a small percentage only of Oriental women have any profession. The population was accordingly divided into six categories as follows:

1. Professional workers (scientists, technologists, managers and clerks).
2. Salesmen.
3. Small farm owners, transportation and skilled workers.
4. Public service workers.
5. Unskilled workers.
6. Unknown.

Interviews of the control women were usually carried out at their place of residence. Some were conducted at nurseries and Health Insurance clinics in collaboration with public and medical local nurses who were acquainted to the interviewed. The interviews were carried out in the interval of 2 years (1963–65).

The groups under study were matched by a special questionnaire for age, country of origin of the subject, education, profession and profession of the husband, ethnic derivation and marital status. Other questions concerned physiological and endocrine factors which included menarche, menstrual disturbances, menopause and menopausal disturbances, family history, chronic diseases and previous medical treatments (hormonal, operations *etc.*).

Data on the patients were obtained from records of the hospitals, from mammary disease clinics and surgical departments. When necessary, the data were completed from the records of pathology and radiology departments and from follow-up controls of the patients (MacMahon *et al.*, 1960).

The data were coded on IBM cards and analysed statistically (chi square "$\chi_2$" and "$t$" tests). Differences were considered significant at the level of $P \leqslant 0{\cdot}05$. In view of results obtained in a previous study (Bertini and Ber, 1964), and as already mentioned, special emphasis in the statistical analysis of the results was given to population of Oriental background, particularly to two Asian groups, Iraqis and Yemenites, differing strikingly in their environmental conditions and in the degree of their adaptation to the Israeli mode of life, aiming at a selective epidemiological demonstration of the importance of the factors investigated.

### RESULTS

Of a total of 13,718 Jewish women included in this study 44·2% were of Oriental origin, 19·8% Israeli-born and 36·0% of Western origin (Table I).

TABLE I.—*Percentage Distribution of Patients and Controls According to Country of Origin*

| Country of origin | Total | Benign mastopathy | Breast cancer | Controls |
|---|---|---|---|---|
| All countries: Abs. No. | 13,718 | 1816 | 1298 | 10,604 |
| % | 100·0 | 100·0 | 100·0 | 100·0 |
| Western | 36·0 | 68·5 | 86·6 | 24·2 |
| Israel | 19·8 | 11·0 | 3·2 | 23·4 |
| Oriental | 44·2 | 20·5 | 10·2 | 52·4 |
| Yemen | 5·8 | 2·4 | 1·0 | 7·0 |
| Iraq | 12·2 | 3·8 | 3·5 | 14·7 |
| Other Asians* | 10·2 | 2·6 | 1·1 | 12·7 |
| African† | 16·0 | 11·7 | 4·6 | 18·0 |

* Iran, Syria, Turkey, India.

† Morocco, Tunisia, Algeria, Libya, Egypt.

TABLE II.—*Percentage Distribution of Patients and Controls According to Age and Country of Origin*

| | Country of origin | | | | | | | | |
|---|---|---|---|---|---|---|---|---|---|
| | Total | Western | Israel | Oriental | | | | | |
| | | | | Total | Yemen | Iraq | Other Asian | African |
| | | Benign mastopathy | | | | | | | |
| Absolute No. | 1816 | 1245 | 199 | 372 | 43 | 69 | 47 | 213 |
| Percentage | 100·0 | 100·0 | 100·0 | 100·0 | 100·0 | 100·0 | 100·0 | 100·0 |
| Age group | | | | | | | | |
| –12 | 1·0 | 0·1 | 8·0 | 0·3 | — | — | — | 0·5 |
| 13–16 | 1·0 | 0·7 | 3·0 | 1·1 | 2·3 | 2·9 | — | 0·5 |
| 17–19 | 2·0 | 0·8 | 5·0 | 4·3 | 2·3 | 2·9 | 4·3 | 5·2 |
| 20–29 | 15·1 | 6·6 | 30·2 | 35·8 | 27·9 | 8·7 | 40·4 | 45·0 |
| 30–39 | 22·5 | 19·6 | 30·2 | 28·2 | 23·3 | 36·2 | 23·4 | 27·7 |
| 40–54 | 49·3 | 60·4 | 23·1 | 25·5 | 39·6 | 43·5 | 25·5 | 16·9 |
| 55–64 | 6·8 | 8·7 | 0·5 | 4·0 | 2·3 | 5·8 | 2·1 | 9·2 |
| 65– | 2·3 | 3·1 | — | 0·8 | 2·3 | — | 4·3 | — |
| | | Breast cancer | | | | | | |
| Absolute No. | 1298 | 1124 | 42 | 132 | 13 | 45 | 14 | 60 |
| Percentage | 100·0 | 100·0 | 100·0 | 100·0 | 100·0 | 100·0 | 100·0 | 100·0 |
| Age group | | | | | | | | |
| 13–16 | 0·2 | 0·2 | — | — | — | — | — | — |
| 17–19 | 0·1 | — | 2·4 | — | — | — | — | — |
| 20–29 | 1·9 | 1·2 | 9·5 | 6·1 | 7·7 | 8·9 | — | 5·0 |
| 30–39 | 12·1 | 10·1 | 28·6 | 24·1 | 15·4 | 24·4 | 28·6 | 25·0 |
| 40–54 | 53·2 | 54·6 | 38·1 | 46·2 | 53·8 | 40·0 | 64·3 | 45·0 |
| 55–64 | 21·9 | 22·8 | 14·3 | 16·7 | 15·4 | 20·0 | 7·1 | 16·7 |
| 65– | 10·6 | 11·1 | 7·1 | 6·8 | 7·7 | 6·7 | — | 8·3 |
| | | Controls | | | | | | |
| Absolute No. | 10,604 | 2561 | 2479 | 5564 | 744 | 1563 | 1345 | 1912 |
| Percentage | 100·0 | 100·0 | 100·0 | 100·0 | 100·0 | 100·0 | 100·0 | 100·0 |
| Age group | | | | | | | | |
| 13–16 | 0·9 | — | 0·3 | 1·6 | 1·8 | — | 1·5 | 3·0 |
| 17–19 | 4·4 | 3·2 | 5·7 | 4·3 | 1·5 | 5·8 | 3·4 | 4·9 |
| 20–29 | 47·3 | 31·5 | 58·6 | 49·7 | 48·7 | 50·4 | 50·2 | 48·9 |
| 30–39 | 27·4 | 28·3 | 26·9 | 27·1 | 32·0 | 25·9 | 28·8 | 25·1 |
| 40–54 | 14·3 | 25·5 | 6·3 | 12·7 | 12·0 | 14·6 | 11·9 | 12·1 |
| 55–64 | 4·3 | 8·5 | 2·2 | 3·2 | 2·4 | 2·3 | 3·1 | 4·3 |
| 65– | 1·4 | 3·0 | — | 1·4 | 1·6 | 1·0 | 1·1 | 1·7 |

The age distribution in the whole population under study was as follows: 58·4% of the women with benign mastopathy and 85·7% of the breast cancer patients were older than 40, while in the control group only 20% were of this age (Table II). This distribution led us to select and investigate separately the three age groups 30–39, 40–54, and 55–64 in order to allow the drawing of conclusions of statistical significance.

The data showed that 75·1% of the breast cancer patients were aged 40–64 (two-thirds of them 40–54 years old), whereas only 14·3% of these patients were younger than 40 and 10·6% older than 65.

Among the benign mastopathy patients of Oriental origin 42·7% were Asians and 57·3% Africans. Among the 159 patients of Asian origin 27% were Yemenites, 43·4% Iraqis and 29·6% from other Asian countries.

Of epidemiological interest is the fact that the Orientals and Israeli-born represented percentages of the total benign mastopathy cases (20·5% and 11·0% respectively) which were higher than those represented out of the total number of breast cancer cases (10·2% and 3·2%). By contrast, the women of Western origin represented 68·5% of the total benign mastopathy cases as against 86·6% of the total breast cancer cases.

*Education.*—The data presented in Table III show that in all the groups under study educational levels were considerably higher in women of Western origin and in Israeli-born than in women of Oriental origin. The percentages of Westerners with 9 years education were in all three groups investigated (cancer, benign mastopathy and control) statistically higher than in Eastern women.

TABLE III.—*Percentage Distribution of Patients and Controls According to Origin and to Years of Schooling*

| | Total | | Benign mastopathy | | Breast cancer | | Controls | |
|---|---|---|---|---|---|---|---|---|
| | Western | Oriental | Western | Oriental | Western | Oriental | Western | Oriental |
| Absolute No. | 7650 | 6068 | 1444 | 372 | 1666 | 132 | 5040 | 5564 |
| Percentage | 100·0 | 100·0 | 100·0 | 100·0 | 100·0 | 100·0 | 100·0 | 100·0 |
| Years of Schooling | | | | | | | | |
| 0 | 4·8 | 24·1 | 3·1 | 17·2 | 2·2 | 18·9 | 5·9 | 24·7 |
| 1–8 | 28·4 | 54·0 | 23·5 | 52·5 | 20·1 | 56·8 | 31·7 | 54·0 |
| 9–12 | 53·2 | 20·4 | 56·1 | 26·8 | 59·4 | 22·3 | 50·9 | 19·9 |
| 12– | 13·6 | 1·5 | 17·2 | 3·5 | 18·3 | 2·5 | 11·5 | 1·4 |

When the selected age groups were compared (Table IV) it was found that in all the age groups of both patients and controls the percentage of women with more than 9 years education was significantly higher ($P < 0{\cdot}001$) in Westerners and Israeli-born than in the Orientals*. In Westerners, in all the selected age groups, the percentage of women with more than 9 years' education was significantly higher ($P < 0{\cdot}001$) in patients than in controls and higher in cancer patients than in benign mastopathy patients ($P < 0{\cdot}01$–$0{\cdot}05$). In Orientals the same trend existed but the differences were not statistically valid. The only significant differences ($P < 0{\cdot}01$–$0{\cdot}02$) were found in the age groups 30–39 and 40–54 in which the percentages of women with more than 9 years of education in the benign mastopathy patients were higher than in controls.

* Since no differences were found between Westerners and Israeli-born they were combined in Tables IV and VII under the heading: Western.

TABLE IV.—*Percentage Distribution of Patients and Controls in Selected Age Groups According to Origin and to Years of Education*

| | Westerners | | | | | | Orientals | | | | | |
|---|---|---|---|---|---|---|---|---|---|---|---|---|
| | Total | | Years of schooling | | | | Total | | Years of schooling | | | |
| Age groups | Absolute numbers | % | 0 | 1–8 | 9–12 | 12– | Absolute numbers | % | 0 | 1–8 | 9–12 | 12– |
| | | | | | Benign mastopathy | | | | | | | |
| 30–39 | 304 | 100·0 | 2·3 | 19·4 | 54·9 | 23·4 | 105 | 100·0 | 11·4 | 50·5 | 33·3 | 4·8 |
| 40–54 | 798 | 100·0 | 2·8 | 27·7 | 54·0 | 15·5 | 95 | 100·0 | 22·1 | 54·7 | 21·1 | 2·1 |
| 55–64 | 109 | 100·0 | 9·2 | 31·2 | 45·8 | 13·8 | 15 | 100·0 | 26·7 | 53·3 | 20·0 | — |
| | | | | | Breast cancer | | | | | | | |
| 30–39 | 125 | 100·0 | 1·6 | 12·0 | 60·8 | 25·6 | 32 | 100·0 | 9·4 | 50·0 | 37·5 | 3·1 |
| 40–54 | 631 | 100·0 | 1·9 | 20·9 | 59·1 | 18·1 | 61 | 100·0 | 21·3 | 59·0 | 16·4 | 3·3 |
| 55–64 | 262 | 100·0 | 3·1 | 24·4 | 57·2 | 15·3 | 22 | 100·0 | 27·3 | 59·1 | 13·6 | — |
| | | | | | Controls | | | | | | | |
| 30–39 | 1392 | 100·0 | 4·0 | 27·8 | 55·9 | 12·3 | 1510 | 100·0 | 20·0 | 52·9 | 25·0 | 2·1 |
| 40–54 | 809 | 100·0 | 7·4 | 34·7 | 46·8 | 11·1 | 708 | 100·0 | 32·3 | 55·4 | 11·9 | 0·4 |
| 55–64 | 273 | 100·0 | 11·0 | 45·8 | 34·8 | 8·4 | 178 | 100·0 | 34·3 | 56·7 | 9·0 | — |

When the Oriental ethnic groups were compared it was found that among Iraqi women of the control group 10·4% were illiterate as compared to 43·8% among Yemenites (Table V). In the cancer group Iraqis without formal education represented a percentage of 6·7 as against 30·8% respresented by the Yemenites. Among the 16 Oriental patients with mammary diseases and with an education of 12 years (equivalent to college) or more, Iraqis represented 66% while in the controls with the same educational level only 52·6%. It is of interest in this connection that the Iraqi women represented 22·6% of the total number of Oriental patients and 28·1% of the total number of Oriental controls.

*Socio-economic factor.*—According to the husband's occupation, 44·8% of the total cases of breast cancer and 48·9% of the benign mastopathies may be classed in category 1 (professionals, scientists, managers, clerks) as against 28·4% of the control group. By contrast, only 12·2% of cancer cases and 9·7%

TABLE V.—*Percentage Distribution of Oriental Patients and Controls According to Country of Origin and to Years of Schooling*

| | | Total | | Years of schooling | | | |
|---|---|---|---|---|---|---|---|
| | Country of origin | Absolute numbers | % | 0 | 1–8 | 9–12 | 12– |
| Benign mastopathy | Total | 372 | 100·0 | 17·2 | 52·5 | 26·8 | 3·5 |
| | Yemen | 43 | 100·0 | 30·3 | 48·8 | 18·6 | 2·3 |
| | Iraq | 69 | 100·0 | 4·3 | 43·6 | 42·0 | 10·1 |
| | Other Asian | 47 | 100·0 | 17·0 | 42·6 | 38·3 | 2·1 |
| | African | 213 | 100·0 | 18·8 | 58·2 | 21·1 | 1·9 |
| Breast cancer | Total | 132 | 100·0 | 18·9 | 56·8 | 22·0 | 2·3 |
| | Yemen | 13 | 100·0 | 30·8 | 53·8 | 15·4 | — |
| | Iraq | 45 | 100·0 | 6·7 | 44·4 | 42·2 | 6·7 |
| | Other Asian | 14 | 100·0 | 28·6 | 57·1 | 14·3 | — |
| | African | 60 | 100·0 | 23·3 | 66·7 | 10·0 | — |
| Controls | Total | 5564 | 100·0 | 24·7 | 54·0 | 19·9 | 1·4 |
| | Yemen | 744 | 100·0 | 43·8 | 44·5 | 10·6 | 1·2 |
| | Iraq | 1563 | 100·0 | 10·4 | 43·3 | 43·6 | 2·7 |
| | Other Asian | 1345 | 100·0 | 21·0 | 67·5 | 10·6 | 0·9 |
| | African | 1912 | 100·0 | 31·5 | 57·0 | 10·7 | 0·8 |

of mastopathy cases belonged to category 5 (unskilled workers) as against 22·3% of controls (Table VI).

Among breast cancer patients of Western origin 46·3% as against 40% of the controls may be classed in category 1 and only 10·8% and 12·2% respectively in category 5. These differences were more marked in the Oriental population, in which 31·8% of the breast cancer patients belonged to category 1 and 24·3 % to category 5, while in the control group 18% belonged to category 1 and 31·3% to category 5 (Table VI).

TABLE VI.—*Percentage Distribution of Married Patients and Controls According to Origin and to Husband's Occupation*

| Group | Husband's occupation | Total Western | Total Oriental | Benign mastopathy Western | Benign mastopathy Oriental | Breast cancer Western | Breast cancer Oriental | Controls Western | Controls Oriental |
|---|---|---|---|---|---|---|---|---|---|
| | Absolute numbers | 7405 | 5940 | 1381 | 369 | 1163 | 132 | 4861 | 5439 |
| | Percentage | 100·0 | 100·0 | 100·0 | 100·0 | 100·0 | 100·0 | 100·0 | 100·0 |
| 1 | Professionals (white collar) | 43·6 | 19·1 | 54·0 | 29·8 | 46·3 | 31·8 | 40·0 | 18·0 |
| 2 | Salesmen | 3·9 | 7·2 | 6·9 | 11·7 | 4·7 | 12·9 | 3·0 | 6·8 |
| 3 | Small farm owners, transportation and skilled workers | 31·1 | 34·4 | 25·7 | 29·5 | 25·8 | 23·5 | 33·9 | 35·0 |
| 4 | Public workers | 7·9 | 7·0 | 5·6 | 4·3 | 7·1 | 4·5 | 8·7 | 7·3 |
| 5 | Unskilled workers | 11·0 | 30·5 | 6·5 | 21·4 | 10·8 | 24·3 | 12·2 | 31·3 |
| 6 | Unknown | 2·5 | 1·8 | 1·3 | 3·3 | 5·3 | 3·0 | 2·2 | 1·6 |

It should be noted that in all three groups investigated (cancer, benign mastopathy, controls) the percentage of Western women belonging to category 1 was significantly higher than that of Orientals, while the opposite was true for those belonging to category 5. In both ethnic groups the percentage of patients belonging to category 1 was significantly higher than that of the controls. In the Western population the percentage of women in category 1 was significantly higher in the group of benign mastopathy patients than in the cancer patients while no statistically valid differences were found in this respect between the two kinds of patients in the Oriental population.

When selected age groups were compared, it was found (Table VII) that in both patients and controls and in all the age groups investigated the percentage of Western women belonging to category 5 was significantly smaller ($P < 0{\cdot}05$–$0{\cdot}001$) than that of Orientals while the opposite was true ($P < 0{\cdot}05$–$0{\cdot}001$) for category 1 with the only exception of cancer patients in the age group 30–39. In Westerners of age groups 30–39 and 40–54 the percentage of women belonging to category 1 was significantly ($P < 0{\cdot}05$–$0{\cdot}001$) higher in patients than in controls, while in the age group 55–64 only the difference between cancer patients and controls was significant ($P < 0{\cdot}05$). In the Orientals the percentage of women belonging to category 1 was much higher ($P < 0{\cdot}001$) in patients than in controls in age groups 30–39 and 40–54, while in the oldest age group the differences in this respect were not significant. Among Westerners in all the selected age groups the percentage of women belonging to category 5 was significantly higher in controls than in benign mastopathy patients ($P < 0{\cdot}001$) but the differences between controls and cancer patients were in this respect non-significant. In the Orientals belonging to category 5 significant differences ($P < 0{\cdot}05$–$0{\cdot}001$) were found only in the

TABLE VII.—*Percentage Distribution of Married Patients and Controls in Selected Age Groups According to Origin and to Husband's Occupation*

| | | Westerners | | | | | | | | | Orientals | | | | | | | | |
|---|---|---|---|---|---|---|---|---|---|---|---|---|---|---|---|---|---|---|---|
| | | Total | | Husband's occupation group | | | | | | Total | | Husband's occupation group | | | | | |
| | Age group | Absolute numbers | % | 1 | 2 | 3 | 4 | 5 | 6 | Absolute numbers | % | 1 | 2 | 3 | 4 | 5 | 6 |
| Benign | 30–39 | 299 | 100·0 | 59·9 | 5·6 | 19·4 | 10·7 | 3·7 | 0·7 | 105 | 100·0 | 35·2 | 6·7 | 34·3 | 3·8 | 19·0 | 1·0 |
| mastopathy | 40–54 | 795 | 100·0 | 53·5 | 6·9 | 27·2 | 3·0 | 8·1 | 1·3 | 95 | 100·0 | 26·3 | 14·7 | 25·3 | 5·3 | 24·2 | 4·2 |
| | 55–64 | 102 | 100·0 | 40·2 | 10·8 | 31·4 | 10·8 | 2·9 | 3·9 | 15 | 100·0 | 13·3 | 26·7 | 20·0 | 6·7 | 20·0 | 13·3 |
| Breast cancer | 30–39 | 125 | 100·0 | 52·0 | 4·0 | 20·0 | 9·6 | 8·0 | 6·4 | 32 | 100·0 | 40·6 | 6·3 | 28·1 | 3·1 | 21·9 | — |
| | 40–54 | 631 | 100·0 | 47·5 | 2·7 | 28·3 | 5·5 | 11·1 | 4·9 | 61 | 100·0 | 31·1 | 14·8 | 21·3 | 4·9 | 24·6 | 3·3 |
| | 55–64 | 262 | 100·0 | 40·5 | 9·9 | 23·3 | 9·5 | 11·1 | 5·7 | 22 | 100·0 | 18·2 | 18·2 | 22·7 | 4·5 | 27·3 | 9·1 |
| Controls | 30–39 | 1377 | 100·0 | 43·0 | 1·5 | 34·4 | 10·2 | 10·6 | 0·3 | 1499 | 100·0 | 23·0 | 5·2 | 39·9 | 8·0 | 23·3 | 0·6 |
| | 40–54 | 799 | 100·0 | 37·4 | 4·8 | 35·8 | 5·1 | 14·0 | 2·9 | 701 | 100·0 | 10·1 | 9·1 | 27·1 | 5·4 | 46·9 | 1·4 |
| | 55–64 | 270 | 100·0 | 32·1 | 5·5 | 25·6 | 11·9 | 15·3 | 9·6 | 176 | 100·0 | 6·8 | 11·4 | 25·0 | 9·1 | 37·5 | 10·2 |

age group 40–54 although the trend for a higher percentage in controls than in patients was noted also in the other selected age groups.

Of special interest are the data concerning socio-economic factors in individual ethnic groups of Oriental origin, particularly in Iraqis and Yemenites (Table VIII). Among the Oriental breast cancer patients belonging to category 1 61·9% were Iraqis while Yemenites represented only 2·4% in this category, as compared to 9·8% of the total population of Oriental breast cancer patients.

TABLE VIII.—*Percentage Distribution of Married Oriental Patients and Controls According to Country of Origin and to Husband's Occupation*

| | Country of origin | Absolute numbers | % | Husband's occupation group* | | | | | |
|---|---|---|---|---|---|---|---|---|---|
| | | | | 1 | 2 | 3 | 4 | 5 | 6 |
| Benign mastopathy | Total | 369 | 100·0 | 29·8 | 11·7 | 29·5 | 4·3 | 21·4 | 3·3 |
| | Yemen | 43 | 100·0 | 4·7 | — | 51·2 | 2·3 | 41·8 | — |
| | Iraq | 69 | 100·0 | 50·7 | 2·9 | 10·1 | 8·7 | 23·2 | 4·4 |
| | Other Asian | 46 | 100·0 | 32·6 | 8·7 | 28·3 | 4·3 | 23·9 | 2·2 |
| | African | 211 | 100·0 | 27·5 | 17·5 | 31·8 | 3·3 | 16·1 | 3·8 |
| Breast cancer | Total | 132 | 100·0 | 31·8 | 12·9 | 23·5 | 4·5 | 24·3 | 3·0 |
| | Yemen | 13 | 100·0 | 7·7 | — | 46·1 | 7·7 | 38·5 | — |
| | Iraq | 45 | 100·0 | 57·8 | 4·4 | 11·1 | — | 20·0 | 6·7 |
| | Other Asian | 14 | 100·0 | 14·3 | 14·3 | 21·4 | 7·1 | 42·9 | — |
| | African | 60 | 100·0 | 21·7 | 21·7 | 28·2 | 6·7 | 20·0 | 1·7 |
| Controls | Total | 5439 | 100·0 | 18·0 | 6·8 | 35·0 | 7·3 | 31·3 | 1·6 |
| | Yemen | 721 | 100·0 | 7·1 | 1·7 | 43·3 | 4·0 | 42·1 | 1·8 |
| | Iraq | 1527 | 100·0 | 29·9 | 4·0 | 28·1 | 7·7 | 28·5 | 1·8 |
| | Other Asian | 1321 | 100·0 | 13·6 | 5·2 | 34·0 | 6·0 | 39·0 | 2·2 |
| | African | 1870 | 100·0 | 15·6 | 12·2 | 38·2 | 9·0 | 24·0 | 1·0 |

* For designation of groups see Table V.

Among the Oriental patients with benign mastopathy 29·8% belonged to category 1. The Iraqis represented 31·8% of the total 110 cases in this category and only 18·7% of the total benign mastopathy cases. These differences were statistically highly significant ($P < 0\cdot001$). In distinction, the Yemenites represented 11·7% of the total benign mastopathy patients of Oriental origin and only 1·8% of those belonging to category 1.

### DISCUSSION

The number of comparative epidemiological studies carried out so far between various immigrant and population groups is very limited. This is regrettable since they could considerably add to our knowledge on the role played by the various constitutional factors in breast cancerogenesis.

The female population groups investigated in this study represented an epidemiological sample of international value by their geographical, racial, ethnic and socio-economic composition. This population consisted of women originating from various continents, tens of countries and a great variety of ethnic communities strikingly differing in their socio-economic and cultural backgrounds as well as in their traditions, customs and habits including religious and nutritional ones. In addition, their places of residence consisting in cities, urban settlements or villages are scattered throughout the country.

The significant differences in the incidence rates of breast disease between women of Eastern and Western origin, between two ethnical groups of Oriental origin—Iraqis and Yemenites, and between two generations, raise a special interest in a study of the environmental conditions of population groups of low breast disease susceptibility in a country in which the average incidence is relatively high. Investigations of this nature may direct attention to the problem whether environmental factors or constitutional properties could be related to breast cancer risk variations.

The two Oriental subgroups, Iraqis and Yemenites, differ widely in their cultural and economic status and in their disease incidence rates. Iraqi women generally belong to higher socio-economic strata and their process of adaptation to the Western way of life is considerably faster than of other women of Asian origin. By contrast, the Yemenites of the first immigrant generation have progressed very little socially and culturally, preserving their habits and traditions, including the religious ones, of the Jews in their country of origin. Their distinctive nutritional habits also remained almost unchanged, their food consisting mainly of vegetables, fruits, grains, spices and vegetable oil. They use almost no animal fats and their diet is relatively poor in proteins.

According to Wynder *et al.* (1960), Wynder and Kaufman (1966), and to Buell and Dunn (1965) there is a relationship between low breast cancer incidence and dietary habits in Japanese women. This hypothesis is supported by the increase in breast cancer incidence among the Japanese immigrants in California who adopted progressively the American diet patterns.

In Israel, nutritional habits are the most obstinately held by most of the ethnic communities. In the present study we investigated the dietary patterns of a few only Oriental groups of women of the first immigrant generation whose breast cancer disease is considered to be the lowest in the country (Bertini and Ber, 1964; Kallner, 1965; Steinitz, 1967). In view of the importance of this factor it should be of interest to study the dietary differences between the other ethnic groups in relation to their breast cancer morbidity.

Naturally, it can be expected that with time the living conditions among Yemenites will change in their process of adaptation to the cultural and economic standards of the country. The second generation will thus be living under completely different conditions and, therefore, a separate investigation of the possible effect of the environmental changes in this group, as well as in other Eastern ethnic groups, on the breast cancer incidence rate seems imperative.

The data of Kallner (1965) and of Steinitz (1967) already point to a significantly faster increase in breast cancer mortality rates in recent years in the Oriental population than in the Western.

Of special epidemiological significance is the similarity in the differences in educational and socio-economic status between groups of high and low breast cancer susceptibility, between Westerners and Orientals, between the old and new generations, between Iraqis and Yemenites and between mammary disease patients and controls in all the groups investigated.

All the Oriental groups with a low incidence of breast disease are of low educational standards. A relatively high percentage of the women of the first generation in these groups are still illiterate or lack a regular education. Furthermore, most of the young women possess only a grammar school education (a very small minority only of them attended high school).

The differences in socio-economic conditions between the Western and Eastern populations are also striking. Most of the women in the latter population group belong to category 5 (unskilled workers). By contrast, of higher socio-economic levels (parallel to cultural and educational levels) is the population with a high breast cancer risk, namely, Westerners, Iraqis, and the second generation of immigrants of Eastern origin, as well as the breast disease patients. These differences were found to be statistically highly significant. Similar results were reported by Khanolkar (1961) who determined breast cancer frequencies among various socio-economic groups in India, and by Segi (1957) in Japan. These countries are known for their particularly low breast cancer incidence. Comparable observations were communicated also by Laurent *et al.* (1964) in Iraq, by Stocks (1955) in England and by Schwartz *et al.* (1958) in France.

Examination of the geographical distribution of the places of residence of breast cancer and benign mastopathy patients, as judged from the place of the hospitals from which the data were collected, showed that in the two hospitals of the central area of the country with a total capacity of 993 beds there were four and a half times more cases of breast cancer and 20 times more benign mastopathies than in the hospitals of the South whose total capacity is only smaller by a third (653 beds). In the hospitals of the north there were also proportionately more cases of mammary cancer than in the south. It should be noted in this context that at the time this study was carried out the Orientals constituted about 22% of the population of the central and northern parts of the country as compared to 37·7% of that in the south.

Staszewski (1964) reported a particularly low incidence of mammary disease in the rural communities of Poland in which the socio-economic status of their members was generally low. It should be underlined that in Israel most of the women of Eastern origin of the first generation of immigrants are living in rural communities. By contrast, the majority of the patients (88%), regardless of ethnic origin. live in cities. However, the differences in living conditions between urban and rural communities are less pronounced in Israel than in other countries, possibly on account of the short distances separating the communities and to the specific social architecture of this country.

This study was supported in part by a grant from Kupath-Holim.

The authors are indebted to Dr. David Allalouf for his aid in the preparation of the manuscript.

### REFERENCES

AZAR, H. A.—(1962) *Cancer, N.Y.*, **15**, 66.
BERTINI, B. AND BER, A.—(1964) *Cancer, N.Y.*, **17**, 438.
BUELL, PH. AND DUNN, J. E.—(1965) *Cancer, N.Y.*, **18**, 656.
CHAKLIN, A. V.—(1962) *Bull. Wld Hlth Org.*, **27**, 337.
DOLL, R., PAYNE, P. AND WATERHOUSE, J.—(1966) ' Cancer Incidence in Five Continents " Publication of the International Union against Cancer. Berlin (Springer-Verlag).
DORN, H. F. AND CUTLER, S. J.—(1955) *Publ. Hlth Monogr.*, No. 29.
DUNHAM, L. J. AND DORN, H. F.—(1955) *Schweiz. Z. allg. Path. Bakt.*, **18**, 472.
FINNISH CANCER REGISTER.—Cancer Incidence in Finland, 1953–56.
GRAHAM, S., LEVIN, M. L., LILIENFELD, A. M. AND SHEEHE, P. R.—(1963) *Cancer, N.Y.*, **16**, 13.
HAENSZEL, W. M.—(1962) *J. natn. Cancer Inst.*, **26**, 37.
HIRAYAMA, T. AND WYNDER, E. L.—(1962) *Cancer, N.Y.*, **15**, 28.
HUEPER, W. C.—(1962) *Clin. Pharmac. Ther.*, **3**, 776.

KALLNER, G.—(1965) Cancer Mortality and Morbidity in Israel, 1950–1961. W.H.O. Cancer, 65.
KHANOLKAR, V. R.—(1955) *Schweiz. Z. allg. Path. Bakt.*, **18**, 423.—(1961) *Acta Un. int. Cancr.*, **17**, 903.
LAURENT, C., LEGUERINAIS, J. AND MAUJEL, L.—(1964) Le Cancer au Moyent Orient (III, Iraq). Données epidemiologiques. *Monogr. Inst. natn. d'Hyg.* (Paris) No. 30.
LILIENFELD, A. M.—(1963) *Cancer Res.*, **23**, 1503.
MACMAHON, B., PUGH, T. F. AND IPSEN, J.—(1960) ' Epidemiologic Methods '. Boston (Little, Brown & Co.).
MUIR, G. S.—(1963) *Cancer, N.Y.*, **16**, 812.
NEWILL, V. A.—(1961) *J. natn. Cancer Inst.*, **26**, 405.
SCHWARTZ, D., DENOIX, P. F. AND ROUQUETTE, C.—(1958) *Bull. Ass. fr. Étude Cancer*, **45**, 476.
SEGI, M.—(1955) *Schweiz. Z. allg. Path. Bakt.*, **18**, 668.—(1957) ' Cancer Mortality Statistics in Japan (1953–1955) '. Tohoku University, Public Health Department, Sendai, Japan.
SEGI, M. AND KURIHARA, M.—(1964) ' Cancer Mortality for Selected Sites in 24 Countries, 1960–1961 ', No. 3. Tohoku University Medical School, Sendai, Japan.
SHIMKIN, M. B.—(1963) *J. Am. med. Ass.*, **183**, 358.
STASZEWSKI, J.—(1964) *Br. J. Cancer*, **18**, 1.
STEINITZ, R.—(1967) ' Five Years Morbidity from Neoplasms in Israel's Population Groups, 1960–1964 '. Jerusalem Ministry of Health.
STEWART, H. L., DUNHAM, L. J., CASPER, J., DORN, H. F., THOMAS, L. B., EDGCOMB, J. H. AND SYMEONIDIS, A.—(1966) *J. natn. Cancer Inst.*, **37**, 1.
STOCKS, P.—(1955) *Schweiz. Z. allg. Path. Bakt.*, **18**, 706.
TAYLOR, J. W.—(1963) *Cancer, N.Y.*, **16**, 1530.
WYNDER, E. L., BROSS, I. J. AND HIRAYAMA, T.—(1960) *Cancer, N.Y.*, **13**, 559.
WYNDER, E. L. AND HOFFMAN, D.—(1966) *Med. Clins N. Am.*, **50**, 631.